# The Potential of NIR Spectroscopy and Chemometrics to Discriminate Roast Degrees and Predict Volatiles in Coffee

**DOI:** 10.3390/molecules29020318

**Published:** 2024-01-09

**Authors:** Stella Green, Emily Fanning, Joy Sim, Graham T. Eyres, Russell Frew, Biniam Kebede

**Affiliations:** 1 Department of Food Science, University of Otago, Dunedin 9054, New Zealand; stella.green@postgrad.otago.ac.nz (S.G.); fanem371@student.otago.ac.nz (E.F.); simjo010@student.otago.ac.nz (J.S.); graham.eyres@otago.ac.nz (G.T.E.); 2 Oritain Global Limited, 167 High Street, Dunedin 9016, New Zealand; rfrew@oritain.com

**Keywords:** coffee, roasting, NIR, rapid analysis, volatile, prediction, fingerprinting, GC-MS, chemometrics

## Abstract

This study aimed to establish a rapid and practical method for monitoring and predicting volatile compounds during coffee roasting using near-infrared (NIR) spectroscopy coupled with chemometrics. Washed Arabica coffee beans from Ethiopia and Congo were roasted to industry-validated light, medium, and dark degrees. Concurrent analysis of the samples was performed using gas chromatography-mass spectrometry (GC-MS) and NIR spectroscopy, generating datasets for partial least squares (PLS) regression analysis. The results showed that NIR spectroscopy successfully differentiated the differently roasted samples, similar to the discrimination achieved by GC-MS. This finding highlights the potential of NIR spectroscopy as a rapid tool for monitoring and standardizing the degree of coffee roasting in the industry. A PLS regression model was developed using Ethiopian samples to explore the feasibility of NIR spectroscopy to indirectly measure the volatiles that are important in classifying the roast degree. For PLSR, the data underwent autoscaling as a preprocessing step, and the optimal number of latent variables (LVs) was determined through cross-validation, utilizing the root mean squared error (RMSE). The model was further validated using Congo samples and successfully predicted (with R^2^ values > 0.75 and low error) over 20 volatile compounds, including furans, ketones, phenols, and pyridines. Overall, this study demonstrates the potential of NIR spectroscopy as a practical and rapid method to complement current techniques for monitoring and predicting volatile compounds during the coffee roasting process.

## 1. Introduction

As one of the most popular beverages worldwide, coffee owes much of its appeal to its distinctive aroma, which plays a pivotal role in consumer acceptance [1]. The roasting process is vital in shaping the distinct flavor and aroma profile of coffee [2]. Variety, origin, and processing conditions (such as fermentation) also influence the volatile flavor profile of coffee beans [3]. The degree of roasting (categorized as light, medium, or dark) significantly impacts the type and concentration of volatile flavor compounds generated during roasting. Lighter roasts are known for their abundance of precursor compounds, including sugars, carbonic acids, and green, grassy-associated volatiles such as esters, aliphatic aldehydes, and terpenes [4,5,6]. On the other hand, extended roasting durations facilitate complex chemical transformations, leading to the generation of a wide range of volatile compounds through processes like the Maillard reaction, Strecker degradation, caramelization, hydrolysis, and pyrolysis [7]. Due to these significant transformations, specific flavor characteristics are associated with certain levels of roasting by consumers. For instance, a dark French roast is often linked to astringent, smoky, and dark chocolate notes.

The volatile profile, overall flavor, and extent of roast degree are inherently interconnected, presenting an opportunity to characterize the roast degree of coffee beans based on their volatile compositions. The current methods used in the coffee industry to determine roast degrees lack robustness and consensus. They fail to consider the volatile differences between roast degrees that contribute to specific flavor variations. For example, Agtron values classify beans as light, medium, or dark roasts solely based on the color values assigned to the beans. While Agtron values can offer an indication of roast degree, their accuracy may be compromised by additional pigments in the beans, and they do not consider the volatile flavor aspect in classifying roast degree [8]. Hence, there is a pressing need to develop an efficient, practical, and reliable method for monitoring and standardizing coffee roasting while predicting the volatile changes that occur during the process. Such a method would yield significant benefits for the coffee industry.

One of the most common and well-established methods for analyzing volatile compounds is gas chromatography-mass spectrometry (GC-MS). However, this method is time- and labor-intensive, as well as expensive. This study proposes the utilization of near-infrared (NIR) spectroscopy combined with chemometrics as an alternative and/or complementary approach. This method is rapid, cost-effective, and aims to discriminate roast degrees while predicting volatile compounds for inferences on flavor.

Recent studies have shown NIR’s ability to discriminate coffee based on geographical origin and sensory cupping scores through the accurate prediction of macronutrients, micronutrients, and isotopes [4,9]. The mechanism by which NIR operates is first by light in the 800–2500 nm spectrum inducing a specific frequency and type of bond vibration (C-H, N-H, O-H) [10]. These vibrations are anharmonic and interfere with the light in this NIR region due to reflection and absorption to various extents, generating unique spectra that result in a highly dimensional spectral dataset [11,12]. It is the intramolecular interactions and associated factors such as bond strength, atomic mass, and environment that affect the intensity and frequency of the bond vibrations and signals. It is likely that the presence and abundance of volatiles in the coffee matrix influence the organic compounds that signal in NIR, which is likely responsible for its ability to predict composition [10]. Volatiles have been successfully predicted for coffee samples and wine samples using NIR, although more work is needed to elucidate the exact mechanism by which this occurs [13,14]. Chemometric modeling techniques can be employed to analyze these complex spectral data, uncover patterns, and extract relevant information [15].

For the prediction of quality-related attributes, initial data obtained from well-established and time-/cost-intensive methods such as GC-MS can be used as a validation dataset, providing a reference for NIR analysis [16]. Once the predictive model is established, there is no need for additional destructive data acquisition, enhancing the efficiency and practicality of the proposed method [12].

Recent studies have demonstrated growing interest in the application of NIR coupled with chemometrics for analyzing coffee samples, encompassing areas such as geographical origin discrimination [9,17,18], quality classification [19], monitoring chemical changes during roasting [20], predicting stable isotopes and trace elements [21], and predicting sensory cupping scores [15,22]. While NIR spectra have been employed to predict roast degrees both online and inline, no studies have incorporated initial data from GC-MS to gain additional insights into the volatile composition of different roast degrees, with the ultimate goal of roast degree discrimination.

Although Caporaso et al. [3] utilized initial data from headspace-solid phase microextraction (HS-SPME) GC-MS for hyperspectral imaging, the high equipment cost limits its industry adaptability compared to more practical and standard NIR spectroscopy. Genisheva et al. [14] took a practical approach using NIR analysis, focusing on wine samples and employing ten significant volatile compounds to develop a chemometrics model and simplify the process iteratively. Wu et al. [13] were able to successfully predict 14 coffee volatiles from GC/MS data using NIR rapid methods for fermented and unfermented samples under the lens of wet fermentation’s effect. To our knowledge, no study has explicitly constructed a chemometrics model using rapidly obtained NIR spectra to predict volatile flavor compounds as a function of coffee roasting with initial input from GC/MS.

In light of this research gap and the recognized need for an alternative and/or complementary method to rapidly classify coffee roast degree, incorporating flavor information from volatile composition, the main objective of this study is to establish a chemometrics-driven rapid NIR method for discriminating roast degree and predicting volatile compounds in coffee. Specifically, the study aims to explore the potential of rapid NIR analysis to (i) discriminate between different roasted coffee samples and compare the results with GC-MS analysis and (ii) predict volatile flavor compounds in the differently roasted samples.

## 2. Results and Discussions

### 2.1. Volatile Changes with Roast Degree from HS-SPME-GC-MS Data and Feature Selection

**Figure 1:**
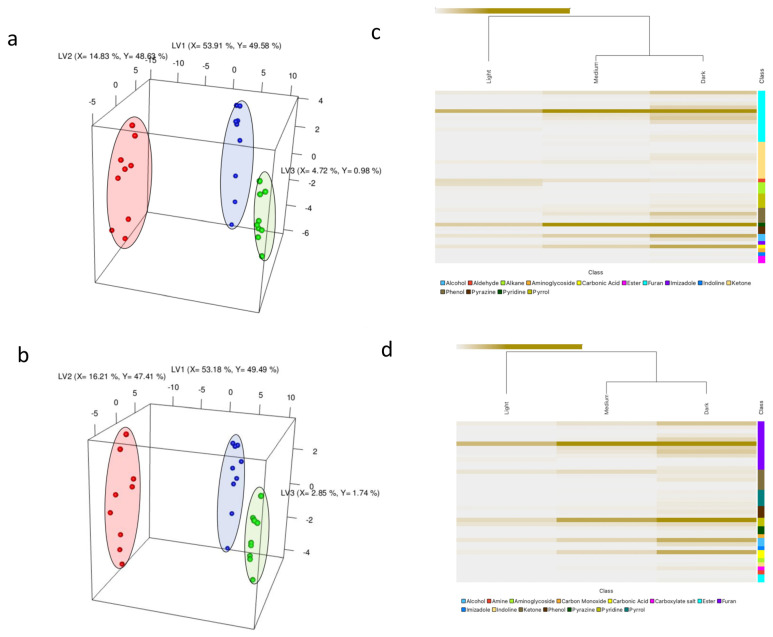
PLS-DA score plot illustrating volatile changes during the roasting process of Arabica coffee beans sourced from Ethiopia (**a**) and Congo (**b**), encompassing nine sample replicates of light (green), medium (blue), and dark (red) roasts. The respective axes indicate the explained variance for each latent variable (LV). Heatmaps showcasing scaled intensities of VID-selected discriminant volatile compounds (>0.9) across light, medium, and dark roasts are presented in (**c**) for Ethiopia and (**d**) for Congo. Intensely colored regions indicate higher compound concentrations.

An unsupervised PCA analysis was initially conducted to explore trends and identify outliers in the volatile compound data. The PCA score plot revealed good reproducibility among replicates, with no outliers identified. Moreover, a clear trend based on the different roast levels was observed. Subsequently, a supervised PLS-DA analysis was performed to investigate the classifications further and identify the volatile compounds contributing to roast degree differentiation (Figure 1). The optimal number of latent variables (LVs) for the model was determined through cross-validation. In this study, two LVs were selected, which resulted in the lowest root mean square error of cross validation (RMSECV) while maximizing the explained variance and minimizing the risk of overfitting.

The PLS-DA analysis successfully discriminated between the different roast degrees, as demonstrated in Figure 1a,b. The separation of the groups along LV1 followed the order of roast degree, with light roast samples positioned on the far right, medium roast samples in the middle, and dark roast samples slightly shifted towards the left. This indicates distinct differences in the volatile composition among the differently roasted samples. These findings are consistent with previous studies showing the discriminative power of GC-MS-based fingerprinting in distinguishing coffee samples based on their roast degree [5,23].

A VID procedure was performed to identify the volatile compounds that had the most significant correlation with the classification of samples into different roast degrees (i.e., discriminant compounds). VID calculates the correlation coefficient between the peak area of volatile compounds and the roast degree. In this study, a correlation threshold of |0.9| was chosen to select the highly discriminating volatiles for roast degree classification. The results were visualized using a heatmap, as shown in Figure 1c,d.

The heatmap analysis revealed that the most abundant classes of compounds, ranked in descending order for dark roasts, were furans, ketones, phenols, pyrroles, esters, pyridines, pyranones, carbonic acids, alcohols, and others. Ketones, furans, imidazoles, and other compounds characterize medium roasts. Light roasts, on the other hand, were characterized by alkanes, aldehydes, alkadienes, and aldehydes. These findings provide valuable insights into the volatile composition of different roast degrees and highlight the specific classes of compounds that can serve as potential markers or indicators, helping to inform the aroma and flavor profiles associated with each roast level.

These volatile trends resulting from roast degrees largely align with the existing literature [3,4,5,6,24]. Therefore, supporting the results of the dataset and establishing its importance when developing a volatile reflective model for roast degree prediction using NIR spectroscopy.

### 2.2. The Potential of NIR and Chemometrics to Monitor Changes during Coffee Roasting

The potential of NIR combined with PLS-DA to monitor changes in coffee samples during roasting was investigated. The NIR spectra were preprocessed and analyzed using chemometric analysis, and the PLS-DA model showed excellent performance. The calibration and cross-validation metrics, including sensitivity, specificity, R^2^, and accuracy, exhibited high values, while the classification error and RMSE were low, further confirming the reliability of the model.

**Figure 2:**
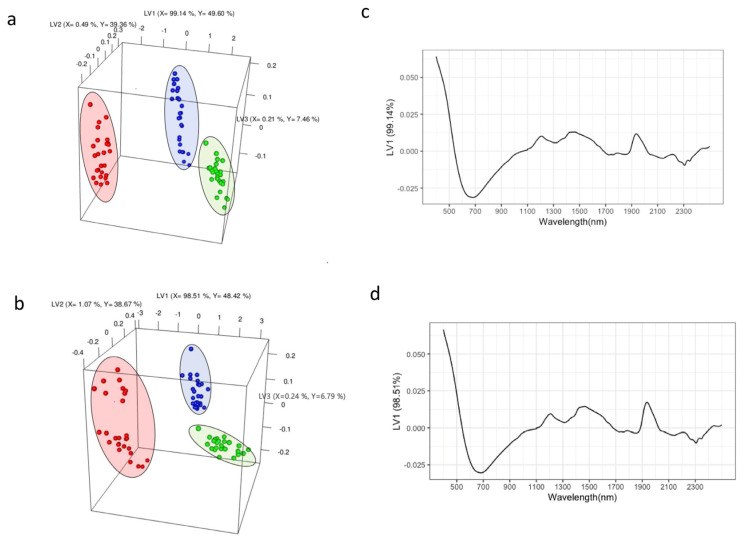
PLS-DA score plot constructed was utilizing the first three latent variables (LVs) showcasing the categorization of roast degrees, encompassing a total of 18 samples from Ethiopia (**a**) and Congo (**b**). Roast degrees are depicted by color: light (green), medium (blue), and dark (red). The loading plots (**c**,**d**) represent loading scores for LV1 along with the corresponding wavelengths (nm).

The PLS-DA score plots based on the NIR spectra for each origin demonstrated clear separation among the different roast degrees (Figure 2a,b). Moreover, the NIR-based PLS-DA model achieved accuracy values of over 0.9 for all roast degree predictions, except for the Congo dark roast sample, which achieved an accuracy of 0.89. This implies an excellent model for roast degree classification, as accuracy considers both error and R^2^. These findings align with a previous study by [25], which used NIR spectra to classify coffee beans based on the industry’s Agtron value roast degree classification. In that study, the authors achieved high R^2^ values for calibration and cross-validation, demonstrating excellent roast degree classification. However, the present study differs in that it does not rely on predicting Agtron values, which can be influenced by color. Another previously published study also reported the ability of NIR combined with chemometrics to classify coffee samples based on roast degree [11]. Alessandrini et al. [11] used different parameters such as weight loss, color, density, and moisture as characteristics of roast degree.

Figure 2c,d illustrates the loadings and associated wavelengths for discriminating the roast degrees. Both geographical origins show similar loading plots for LV1, indicating that the 500–1000 nm wavelength region plays a significant role in the model and is important for differentiating the roast degrees of these coffee samples. This region corresponds to the visible wavelength range (400–800 nm) and includes structural overtones and combinations. The wavelength range of 700–950 nm in the NIR spectrum can be attributed to the 3rd overtone region, which encompasses C-H, H_2_O, R-OH, and R-NH overtone bands [26].

This work demonstrates the significant potential of NIR spectroscopy as a practical and rapid method to complement current methods for monitoring and standardizing the coffee roasting process. Notably, this discrimination pattern closely resembled the differentiation observed in the GC-MS volatile data, indicating that NIR spectra can effectively differentiate between roasted coffee samples. This is likely due to the interactions of coffee volatiles, which may be non-NIR-active, with biomass, which is NIR-active, imposing unique vibrational bond frequencies and modes detected via the reflected wavelengths. Despite these successes, there are several limitations inherent to NIR spectroscopy that need to be discussed. Firstly, the observed classification could be caused by multiple factors in addition to the volatile changes, and it is less straightforward to establish a direct causal relationship. There could also be potential interference from non-NIR-active compounds, and the response depends on the homogeneity and moisture content of the samples.

### 2.3. The Potential of NIR Spectroscopy to Predict Volatile Flavor Compounds

PLS-R models were developed to predict key discriminatory volatile organic compounds (with VID values > 0.8) using NIR spectra as the predictor (X) and the target volatile compound as the response (Y). The models were constructed using Ethiopian samples and subsequently validated using Congo samples.

The performance of the models was assessed based on the coefficients of determination (R^2^) and normalized root mean squared errors (NRMSE) calculated for both the calibration and validation sets. The R^2^ value indicates the goodness of fit between the predicted and actual values, with higher values indicating better model performance for the specific volatile compound. To understand the deviations between actual and predicted values, or how far from the regression line the data points are, the RMSE is normalized to take into account the scale of the response variable. A lower NRMSE value suggests a more accurate and reliable prediction for the volatile compound.

Around 25 volatile organic compounds were successfully predicted with R^2^ values > 0.75 and relatively low NRMSEP for both the calibration and validation sets (Table 1). The predicted compounds spanned various chemical classes, such as ketones, furans, aldehydes, esters, and phenols, and are largely in line with the range of 14 volatiles successfully predicted with NIR by Wu et al. [13]. This indicates that NIR can predict across a broad range of chemical classes that are known to be important discriminators of coffee roast degree.

**Figure 3:**
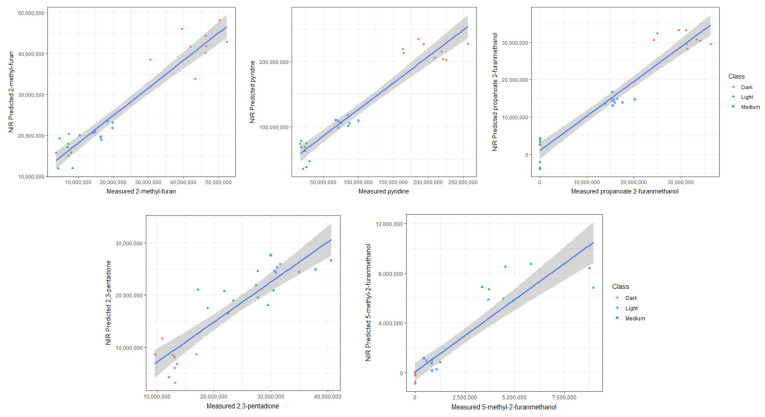
The relationship between peak areas (for representative volatile compounds) and the NIR predicted values. Roast degrees are depicted by color: light (green), medium (blue), and dark (red). The blue lines represent the best-fit line, and the gray ribbons indicate uncertainty intervals.

The chemical classes well predicted in the dark roast samples included furans, ketones, phenols, pyrroles, esters, pyridines, and pyranones. For instance, Figure 3 demonstrates that in dark roasts, 2-methyl-furan and pyridine became prolific, experiencing a significant increase in their relative concentrations compared to light and medium roast degrees. The ester, 2-furanmethanol propanoate, however, increased more gradually with increasing roast degree, as depicted in Figure 3. This progression is linked to the pathways by which they are produced, with more precursor compounds, such as acids and furan alcohols, gradually generated as intermediate products over the course of roasting [27]. These findings align with reported volatile compounds associated with dark-roasted coffee samples, which significantly influence the coffee flavor profile [6]. Compounds like 2,5-dimethylfuran contribute to an ethereal aroma, and pyridine is associated with a bitter and roasted aroma [23].

Far fewer compounds were successfully predicted in the medium roasts. This is because few volatiles reach their maximum levels in medium roasting conditions compared to dark roasting conditions, except for a few intermediate reaction products. Nonetheless, the presence of one key discriminating compound, 2,3-pentadione, was successfully predicted within the specified thresholds, detailed in Table 1 and Figure 3. This ketone is produced through the pyrolysis of carbohydrates and the self-oxidation of lipids and is known for its reported oily/buttery notes [23].

In the light roast samples, the presence of discriminant volatile compounds such as 5-methyl-2-furanmethanol (Figure 3), 2-pentyl-furan, 2,6-dimethyl-2,6-octadiene, and 3-methyl-2-butenoic acid were well predicted using NIR with high R^2^ and low NRMSEP values, as detailed in Table 1.

The NIR signal on roasted coffee samples can be influenced by several confounding factors, such as cultivar, geographical origin, and post-harvest processing conditions, among others. In the present work, only the roasting conditions were varied, and other factors were controlled by ensuring the use of only Arabica, single origin, and wet-washed samples. A second origin was included for validation. Interestingly, when comparing the two origins, there were similarities between the Ethiopian and Congo samples. This is evident in the comparable classification as a function of degree of roasting (PLS score plots) and also in the similarity of discriminant compounds and predicted compounds. Nevertheless, there is a need to investigate the sensitivity of the NIR method and develop models by incorporating more varieties and origins in the future.

In summary, the PLS-R model effectively predicted an increased number of volatile compounds spanning diverse chemical classes, all linked to coffee roast degree in both Ethiopian and Congolese origins. This cross-origin validation further underscores the robustness of the models. However, the underlying reason for the accurate prediction is still not well understood. A proposed mechanism by which NIR is able to predict these roast-degree-associated coffee volatiles is that they are likely non-NIR-active, having an indirect effect on NIR outputs as they influence the signals of NIR-active biomass elements within the coffee complex. Different chemical classes of volatiles share similar bond structures and functionalities within their classes and thus likely affect NIR-active elements in similar modes. As discussed in the introduction, this correlation could alter the bond vibration frequency and type detected in the NIR spectra, thus allowing the volatiles to be predicted. More research is needed to elucidate this mechanism, although this may prove difficult. One approach could be moving into a more targeted approach. The prediction models enable the selection of loadings, which are important wavelengths for the prediction. Perhaps a study narrowing down on a few important wavelengths and then spiking the samples with known/targeted concentrations of discriminant and odor-active volatile compounds.

## 3. Materials and Methods

### 3.1. Sample Sourcing and Storage

Green coffee beans from two geographical origins, namely Congo (catimore and bourbon cultivars) and Ethiopia (heirloom cultivars), were selected for this study. Considering that confounding factors such as cultivar, geographical origin, and post-harvest processing conditions may influence the volatile attributes, attempts were made to control some of these factors by choosing arabica and wet-washed coffee samples. The samples were selected from Ethiopia and Kenya due to their close geographical proximity, with the aim of validating the predictive model on a second geographical origin. The beans were grown at altitudes ranging from 1460 to 2000 m and were sourced from Green Bean House, New Zealand. Before roasting, the beans were carefully inspected to remove any defective or extraneous materials. Subsequently, the selected beans were stored at −20 °C. The selection of beans from the same species, subjected to similar processing conditions, and grown within a similar altitude range aimed to minimize the potential impact of these external factors on the volatile composition under investigation.

### 3.2. Roast Degree Optimization

The optimal time and temperature parameters for the roast degrees were determined based on industry validation from a local Dunedin-based roastery, Common Ground (Unit 114 Strathallan Street, Dunedin 9012, New Zealand). As the objective of the study was to develop a method for the rapid classification of roasted beans based on roast degree, the goal was to select three distinctively different roast degrees from each other, considering the lack of common consensus on what different roast degrees entail even within the industry. Working with the local roastery to best reflect their standardized roast degrees, these were developed using a CBR-101 Innovative Off-axis Rotation Roaster (Gene café, Seoul, Republic of Korea). For consistency and to align with common industry practices, the definition of roast degree was based on specific termination points during the roasting process. A light roast was defined as ending ten seconds after the first crack, a medium roast as terminating ten seconds after the second crack, and a dark roast as continuing 60 s after the second crack, when the beans began to release their oils. Consultation with the roastery validated these roast level stages in the selection of the time and temperature parameters used to achieve them. To better reflect industry processes that rely on time and temperature standardization based on environmental conditions and to mitigate variations in lightness due to additional pigments between origins, standardized time and temperature profiles were employed instead of relying solely on L* values.

Based on these considerations, the final selected time and temperature profiles for roasting were as follows: 9 min at 232 °C for the light roast, 14 min at 232 °C for the medium roast, and 14 min at 250 °C for the dark roast. These parameters were chosen to ensure consistency and to accurately assess the potential of the NIR method. Additionally, they align with standard industry practices for achieving the desired roast degree.

### 3.3. Coffee Bean Roasting, Grinding, and Aliquoting

Samples for HS-SPME, GC-MS, and NIR analysis were prepared in triplicate batches for each roast degree of each origin. Before starting the roast, the roaster was preheated to the target roast temperature and maintained for 6 min. The pre-weighed coffee beans were added to the roaster at the specified time and temperature (see Table S1). To monitor temperature variations across batches, initial and terminal room temperatures, as well as internal roaster temperatures, were recorded using a handheld infrared thermometer (DIGITECH QM7215, Digitech, UT, USA).

After the completion of the roasting process, the roast batches were allowed to cool down, following the instructions provided by the roaster. To facilitate CO_2_ degassing, the uncovered roast batches were left overnight at ambient temperature.

The roasted coffee beans were ground using a Sette 270 grinder (Baratza, Bellevue, WA, USA) set to 14F, achieving approximately 75% of the desired grind size of 400–850 µm. The desired grind size was achieved by sieving the ground coffee through a stack of sieves with openings of 1.6 mm, 850 µm, and 400 µm. Pre- and post-grind measurements of L*, a*, b*, moisture content, and weight loss were recorded (refer to Table S1).

For NIR analysis, each roast batch was transferred to a 5 mL plastic lidded vial, filled to approximately ¾ full, and tightly sealed with aluminum foil to prevent light oxidation. These vials were stored at −20 °C until NIR analysis. For GC-MS analysis, samples were prepared in 20 mL vials, with 3 vials per batch, with each vial containing 1.00 g of ground coffee. This resulted in nine readings for GC/MS per roast level, as this number of replicates has been shown to be sufficient with this time-/cost-intensive technology. Twenty-seven readings were taken for NIR per roast level, as light scattering at the surface of the sample can interfere with the signal, and more readings help mitigate this issue. Moreover, NIR spectra are obtained very quickly and are more cost-effective compared to GC-MS data.

### 3.4. Analysis

#### 3.4.1. HS-SPME-GC-MS Analysis of Volatile Compounds

Headspace volatile analysis was performed using an Agilent Technologies 6890 N gas chromatograph (Agilent Technologies, Santa Clara, CA, USA) equipped with an autosampler (Agilent PAL RSI 85; Palo Alto, CA, USA). Samples were thawed and kept in a cooled tray (6 °C) until analysis. For analysis, samples were transferred to the incubator at 40 °C for 5 min for equilibration with the agitator turned on. The volatile compounds in the headspace were extracted using a divinylbenzene/carboxen/polydimethylsiloxane (DVB/CAR/PDMS) mixed-polarity SPME fiber for 40 min at 40 °C.

For analyte separation, the GC-MS parameters were set as follows: splitless mode for 2 min, constant helium carrier gas flow rate of 1 mL/min, purge flow rate of 50 mL/min for 6 min, followed by a flow rate of 20 mL/min. A ZB wax column with a length of 60 m, an internal diameter of 320 μm, and a film thickness of 0.5 μm was utilized. The GC oven temperature program began at an initial temperature of 50 °C, held for 5 min, followed by a ramping rate of 5 °C/min up to 210 °C, and then increased again at a rate of 10 °C/min to 240 °C with a final hold time of 5 min at this temperature. The mass spectrometer used was a 5975B VL MSD with a triple-axis detector, operating in electron ionization (EI) mode with an energy of 70 eV and scanning in the mass-to-charge ratio (*m*/*z*) range of 29–300.

To minimize potential order effects, the GC-MS vials were randomized during analysis. Blanks and quality control samples were included at the beginning and end of each run to monitor for carryover and assess systematic variations. Standards representative of each chemical class were also analyzed to confirm the identity of volatile compounds, and alkane standards were injected to calculate retention indices (RIs) for compound identification.

#### 3.4.2. NIR Analysis

NIR analysis was performed following the procedure developed by Sim et al. [21] with slight modifications, using a NIRS XDS RapidContent Analyzer (Metrohm, Herisau, Switzerland) equipped with Vision 4.1 software (Metrohm, Herisau, Switzerland). The samples were randomized and analyzed within 6 h of defrosting. NIR spectra were acquired at ambient temperature using a rapid solid module with a spot size of 17.25 mm. A background spectrum was recorded using Spectralon 99% reflectance, including an internal reference standard. A stationary cell and reflectance detector sampling system called a RapidContent solids module (Metrohm, Herisau, Switzerland) were employed, covering a spectrum range of 400–2500 nm with 32 scans per spectrum, a resolution of 5 cm^−1^, and 256 scans.

For each analytical replicate, a 10 mL vial was filled with ground coffee to a level of approximately 1 cm (approximately 2 g powder). Nine analytical replicates were taken for each replicate of the sample to ensure reliable and representative data. The spectra were then preprocessed using extended multiplicative scatter correction (EMSC) and mean centering based on previous optimization work by [21]. Before further analysis, the analytical replicates per replicate were averaged, and the results were saved as text.

### 3.5. Data Analysis

The chemometric analysis was conducted in three distinct stages: (i) analysis of the volatile data, (ii) analysis of the NIR data, and (iii) construction of the PLS-R prediction model. Prior to their respective and integrated analyses, preprocessing steps were applied to the GC-MS chromatograms and NIR spectra to enhance data quality and reduce noise.

#### 3.5.1. Chemometric Analysis of HS-SPME-GC-MS Data

The preprocessing of the total ion chromatograms involved several steps to enhance data quality and reduce noise. First, an automated mass spectral deconvolution and identification system (AMDIS) was used to deconvolute the peaks using the default integration settings. Subsequently, the mass profiler professional (MPP) was applied to align, filter, and remove irregular and non-reproducible peaks. An abundance threshold value of 50,000 was implemented to filter out irregular and noisy peaks. Additionally, non-reproducible peaks, i.e., those not present in at least 80 percent of the replications, were removed. Before the chemometrics data analysis, the final data table was manually cross-checked with the total ion chromatogram to ensure that important volatile compounds were not missed and unwanted peaks (e.g., column bleeds or compounds from the DVB/CAR/PDMS fiber) were not included. This thorough preprocessing step resulted in a total of 166 compounds. The chemometric analysis was performed using SOLO software 9.0 from Eigenvector Research, Inc. (Manson, WA, USA) to reduce the dimensionality of the complex data and identify patterns and discriminant compounds.

The chemometric analysis was conducted stepwise, starting with unsupervised analysis followed by supervised analysis and feature selection, as described by Kebede et al. [28]. Preprocessing included autoscaling, which involved mean centering followed by standardization. Initially, exploratory principal component analysis (PCA) was performed to identify trends, detect outliers, and assess the reproducibility of replicates. Subsequently, a supervised partial least squares discriminant analysis (PLS-DA) was carried out to explore the classification of roast degrees and identify the discriminant volatile organic compounds. The X variables comprised the volatile compounds, while the Y variables comprised the roast degree classes. Cross-validation was performed using the Venetian Blinds method, and the optimal number of latent variables (LV) was selected based on metrics such as the lowest root mean squared error (RMSE) and the maximum variance explained in the data. Score plots were generated to visualize the classification and trends. Separate PLS-DA models were constructed for Ethiopian and Congolese beans in order to compare the classification performance among the samples.

To identify the volatiles driving the classification, feature selection was performed using variable identification (VID), where each volatile compound was assigned a coefficient ranging from −1 to +1. VID scores were calculated using the formula VID = corr(x, ŷ), where x represents the input of volatile abundance and ŷ represents the predicted class by the PLS-DA model. Volatiles with VID scores greater than 0.8 were selected as “key discriminatory markers” for the respective roast degree. The tentative identification of these volatiles was initially achieved using the NIST spectral library, and their identification was further confirmed by injecting standard compounds from each chemical class.

#### 3.5.2. Chemometric Analysis of NIR Spectra

The 162 NIR spectra, comprising 32 scans of 9 replicates for each roast degree batch, were subjected to PLS-DA analysis following an initial exploratory PCA analysis. The individual origins, Congo and Ethiopia, were analyzed separately. NIR spectra were characterized by non-linearities introduced by light scattering. Preprocessing techniques, including extended multiplicative signal correction (EMSC) and mean centering (MC), were applied to the model to address the common NIR spectra artifact of curvature [21]. The construction of the models followed the same methodology described in Section 3.5.1, using the SOLO software 9.0 in Matlab. A PLS-DA classification model was developed to assess the discriminatory potential of NIR in distinguishing between differently roasted samples. Loading plots were employed to identify the regions of the spectra that exhibited significant features for sample classification.

#### 3.5.3. Construction of the PLS-R Model to Predict Volatility

PLS-R models were developed to predict the key discriminatory volatile organic compounds (with VID values > 0.8) using NIR spectra (X-block) as the predictor and the target volatile compounds identified through GC-MS (Y-block). The models were constructed using Ethiopian samples and subsequently validated using Congo samples. To assess the performance of the models, coefficients of determination (R^2^) and normalized root mean squared errors (NRMSEP) were calculated across the calibration and validation sets. These metrics provide insights into the accuracy and reliability of the predictions made by the models.

## 4. Conclusions

This study explored the potential of NIR spectroscopy and chemometrics to discriminate roast degrees and predict volatiles in coffees. By integrating GC-MS data and NIR data through multivariate data analysis, a robust and well-validated model for roast degree classification was developed. The GC-MS models effectively discriminated between different roast degrees, and remarkably, the NIR spectra modeling using PLS-DA achieved a similar level of discrimination. The final NIR volatile prediction model accurately predicted an increased number of volatile compounds spanning diverse chemical classes associated with the roast degree.

The developed method holds significant implications for the coffee industry, enabling roasteries to rapidly predict key coffee volatiles and effectively classify roasts as light, medium, or dark. If applied with handheld NIR equipment, this could help quickly establish roast degree based on perhaps more meaningful flavor information from volatiles as opposed to color-derived indications, providing additional insights into the specific roast batch’s flavor compounds. This standardized approach enhances efficiency and consistency in roast degree classification, while the use of handheld NIR instruments makes it accessible and practical for implementation. Hand-held NIR equipment is also cheaper and less labor-intensive to use, with lower training requirements than the corresponding bulky GC/MS units, making it more attractive to the industry.

In terms of the future applicability of the model developed in this work, there are several limitations. The limited sample size, including only two origins, may make this model struggle to generalize to additional origins, so more need to be included in validation datasets from additional varieties, origins, and post-harvest processing conditions. Another challenge in the implementation of the proposed method is the industry discrepancies between roast levels. Therefore, to further validate the model, additional roasts over a range within each of the roast degrees could be built into the model. Considerations of non-volatile compounds and sensory attributes could be included in the model to provide a more overall insight into the overall flavor. Furthermore, exploring the predictive potential of advanced machine learning models, such as decision trees and artificial neural networks, beyond classical chemometrics methods is essential, especially if a larger and more complex dataset will be investigated.

Overall, this study highlights the potential of NIR spectroscopy combined with multivariate data analysis as a powerful tool for real-time monitoring and prediction of volatile changes during coffee roasting. The findings contribute to improving quality control and standardization in the coffee industry, paving the way for improved product consistency and increased consumer satisfaction. Future work should aim to elucidate the NIR mechanism of detecting volatiles and utilize additional data to increase the robustness of prediction.

## Figures and Tables

**Table 1:** PLS-R model performance metrics based on the coefficients of determination (R^2^) and normalized root mean squared errors (NRMSE) across calibration and validation sets for volatile compounds (VID > 0.8).

Volatile Compound	NRMSEP	R^2^ Pred
2-methyl-furan	0.34	0.92
2,5-dimethyl-furan	0.28	0.94
2,3-pentadione	0.32	0.79
2,6-dimethyl 2,6-octadiene	0.48	0.81
1-methyl-1h-pyrrole	0.61	0.81
pyridine	0.31	0.91
2-pentyl-furan	0.33	0.81
tetrahydro-2-furancarbonyl chloride	0.90	0.77
2-furfurylthiol	0.52	0.93
2-[(methylthio)methyl]-furan	0.83	0.80
2,3-dimethyl-2-cyclopenten-1-one	0.62	0.80
propanoate 2-furanmethanol	0.25	0.92
2,2′-methylenebis-furan	0.45	0.85
4-hydroxy-butanoic acid	0.43	0.84
2-(2-furanylmethyl)-5-methyl-furan	0.53	0.86
5-methyl-2-furanmethanol	1.01	0.82
ethyl 2,3,6,7-tetrahydro-4-oxepinecarboxylate	0.45	0.88
3-methyl-2-butenoic acid	0.42	0.92
1-(2-furanylmethyl)-1h-pyrrole	0.38	0.84
2-methoxy-phenol	0.51	0.77
2,2′-[oxybis(methylene)]bis-furan	0.57	0.84
3-methyl-phenol	1.04	0.75
4-ethyl-2-methoxy-phenol	0.78	0.77
4-methyl-2(1h)-quinolinone	0.78	0.90
cyclopropyl carbinol	0.95	0.80

## Data Availability

The data presented in this study are available on request from the corresponding author. The data are not publicly available due to confidentiality.

## Supplementary Materials

The following supporting information can be downloaded at: https://www.mdpi.com/article/10.3390/molecules29020318/s1. Table S1: Roasted sample bean characterization of roast temperatures, roast time, chromatic values, terminal moisture content, and weight loss percentage for all samples.
