# Supplementary material for: The Potential of NIR Spectroscopy and Chemometrics to Discriminate Roast Degrees and Predict Volatiles in Coffee
Source: Molecules. 2024 Jan 9;29(2):318. doi: 10.3390/molecules29020318 (PMC10820711; doi:10.3390/molecules29020318)
Supplement: Supplementary file 1 [file molecules-29-00318-s001.zip › molecules-2788710-supplementary.pdf]

Table S1

Roasted sample bean characterization of roast temperatures, roast time, chromatic values, terminal moisture content and weight loss percentage for all samples.

Order	Batch	Time (min)	Temp. set (°C)	Air Temp. (°C)	Initial Temp. (°C)	Terminal Temp. (°C)	L* Pre-Ground	a* Pre-Ground	b* Pre-Ground	L* Post-Ground	a* Post-Ground	b* Post-Ground	Moisture Content (%)	Weight Loss (%)
1	Warm up bean	8	250	18.5	119.1±4.0	149.6±4.3	-	-	-	-	-	-	8.08±0.18	11.33
2	Ethiopian Light 1	9	232	19.9	152.0±5.1	174.6±2.0	24.06±0.82	7.66±0.91	14.50±0.89	24.07±0.37	12.55±0.15	23.50±0.15	9.46±0.27	10.67
3	Ethiopian Light 2	9	232	18.4	159.6±4.0	172.8±1.9	25.23±0.55	7.48±0.86	14.74±0.53	24.97±1.24	12.21±0.35	23.11±0.70	9.24±0.37	18.55
4	Ethiopian Light 3	9	232	15.6	161.5±4.2	179.1±4.0	23.62±1.68	7.42±0.58	14.53±0.99	24.54±1.09	12.28±0.20	22.73±0.60	9.22±0.25	11.14
5	Ethiopian Medium 1	14	232	16.7	165.8±1.7	173.2±3.9	20.53±1.58	6.22±0.79	10.91±0.66	16.38±0.84	10.22±0.31	15.63±0.42	7.74±0.15	14.64
6	Ethiopian Medium 2	14	232	17.1	153.5±3.0	173.2±3.1	21.82±1.96	6.92±0.50	11.72±0.89	14.25±0.87	9.56±0.20	14.55±0.42	7.70±0.12	14.28
7	Ethiopian Medium 3	14	232	15.9	149±1.5	178.3±1.0	20.34±0.66	6.66±0.45	11.16±0.57	14.52±0.30	9.78±0.2	15.36±0.57	8.04±0.19	14.07
8	Calibration bean 1	8	250	16.5	161.7±1.5	175.1±1.3	25.36±1.19	9.94±0.32	17.73±0.88	30.64±1.10	11.73±0.21	25.17±0.48	8.16±0.09	11.83
9	Ethiopian Dark 1	14	250	16.8	167.8±2.0	201.5±4.3	16.00±0.67	5.00±0.30	6.71±0.46	7.66±0.24	6.55±0.11	7.23±0.36	6.68±0.16	17.99
10	Ethiopian Dark 2	14	250	17.8	165.4±2.6	201.4±4.8	15.17±1.38	5.23±0.30	7.20±0.94	7.90±0.82	6.84±0.30	6.85±0.84	7.12±0.22	17.49
11	Ethiopian Dark 3	14	250	16.2	161.7±2.3	202.2±2.9	15.98±0.41	5.33±0.55	6.95±0.87	8.62±0.72	7.11±0.28	7.45±0.47	6.88±0.8	17.07
12	Congo Light 1	9	232	17.7	154.0±2.2	173.7±2.4	25.29±0.73	5.52±0.37	13.20±0.40	22.76±1.78	11.22±0.53	21.12±0.91	11.22±0.15	8.49
13	Congo Light 2	9	232	19	164.5±1.2	178.7±1.9	22.51±0.84	6.05±0.56	12.28±0.59	19.16±0.55	10.71±0.22	18.97±0.29	10.33±0.06	10.68
14	Congo Light 3	9	232	18.7	165.0±2.1	176.6±4.0	23.50±2.00	5.52±0.34	12.30±0.77	19.37±1.70	10.68±0.47	18.72±0.63	10.05±0.07	10.7
15	Calibration bean 2	8	250	18.8	162.6±2.0	177.7±2.1	23.98±1.83	9.57±0.42	17.20±0.95	25.19±1.57	12.39±0.39	24.62±0.84	8.16±0.9	9.75
16	Congo Medium 1	14	232	17.6	158.4±1.6	178.3±2.1	19.54±1.81	5.32±0.17	10.54±0.65	13.08±0.65	9.17±0.17	13.46±0.49	8.04±0.09	14.05
17	Congo Medium 2	14	232	22.1	160.4±2.8	179.9±1.0	18.54±0.86	5.53±0.30	9.90±0.73	13.50±0.24	9.48±0.19	13.37±0.25	7.87±0.23	14.43
18	Congo Medium 3	14	232	17.1	163.8±3.3	178.1±1.9	18.51±0.78	5.54±0.20	10.28±0.61	13.34±0.29	9.35±0.24	13.85±0.43	8.30±0.14	13.8
19	Congo Dark 1	14	250	18	164.9±1.6	198.0±2.3	15.12±1.41	4.91±0.24	6.63±0.16	8.45±0.79	6.73±0.24	6.92±0.64	7.70±0.13	17.81
20	Congo Dark 2	14	250	20.5	167.0±2.1	197.3±1.3	14.45±0.72	4.43±0.29	6.19±0.64	8.64±0.28	7.01±0.18	6.94±0.18	6.70±0.17	20.14
21	Congo Dark 3	14	250	16.1	167.0±2.0	193.5±5.5	14.89±1.10	4.65±0.18	6.73±0.70	8.86±0.56	7.09±0.24	7.51±0.44	6.62±0.05	17.65
